# Prevalence of Depressive Disorders in Operatively Treated Pelvic Trauma Patients

**DOI:** 10.3390/diseases13040105

**Published:** 2025-03-31

**Authors:** Piotr Walus, Jakub Ohla, Rafał Wójcicki, Tomasz Pielak, Jakub Bulski, Michał Wesołowski, Gazi Huri, Jan Zabrzyński

**Affiliations:** 1 Department of Orthopaedics and Traumatology, Jan Kochanowski University, 25736 Kielce, Polandjan.zabrzynski@cm.umk.pl (J.Z.); 2 Department of Orthopaedics and Traumatology, Nicolaus Copernicus University, 87100 Toruń, Poland; 3 Department of Pathophysiology, Wroclaw Medical University, 50368 Wrocław, Poland; 4 Department of Orthopaedics and Traumatology, Faculty of Medicine, Hacettepe University, 06800 Ankara, Türkiye

**Keywords:** pelvis, fracture, depression, suicidal ideation, acetabulum

## Abstract

Aim: The aim of this study is to assess the prevalence of depressive disorders in patients with pelvic fractures treated surgically at our center in the years 2017–2022. Materials and Methods: The study included 75 patients, 57 men and 18 women, operated on in our center in the years 2017–2022 due to acetabular fracture and pelvic ring injury. Factors such as age, gender, chronic pain measured with the VAS scale, and the incidence of suicidal thoughts were also analyzed. The participants completed the Beck Depression Inventory (BDI). Results: Thirty-five out of the seventy-five patients showed symptoms of depression (BDI score > 11), which is 47%, with an average score of 29 (*p* < 0.0001). Women obtained an average BDI score of 23, which corresponds to moderate depression on Beck’s scale (*p* < 0.008). The correlation between BDI score and chronic pain in pelvic trauma patients has been found to be positive (*p* < 0.0003; r = 0.4094). Furthermore, women in our studied population reported suicidal thoughts more often than men (44% vs. 14%; *p* < 0.01). No statistically significant correlation was found between the occurrence of depression and the length of hospital stay and the patient’s age (*p* < 0.5 and *p* < 0.06, respectively). Conclusions: The prevalence of depression in the pelvic trauma patients of the studied population has been determined to be 47%.

## 1. Introduction

Depressive disorders are among the most common psychiatric illnesses diagnosed in the 21st century [1,2]. About 3.8% of the population experience depression, including 5% of adults (4% of men versus 6% of women) [1]. The causes of these disorders are usually composed of many important factors. Among them, we can distinguish three main groups: biological, psychological, and social [3]. The biological factors include, for example, accompanying diseases, gender, age, and genetic conditions responsible for the quantity and quality of neurotransmitter production and neural connections [4,5]. The psychological factors include character and temperament, which influence, among other things, the way one copes with life’s difficulties [6]. In the case of social factors, the current life situation and support from third parties play a significant role in the development of depressive disorders [7].

Mental health disorders (MHDs) of orthopedic patients were observed and considered already in the 1970s [8]. Their occurrence in forms such as anxiety, depression, and post-traumatic stress disorder (PTSD) is observed in patients after trauma, and one of the main consequences of this phenomenon includes limited long-term outcomes [9]. It has been observed that, among the adult population, the incidence of depression and PTSD in patients with musculoskeletal injuries is significantly higher [10]. Winter et al. showed that lower-limb fractures carry the risk of new MHDs in previously healthy individuals, with the location of the fracture being important as this risk is higher in fractures proximal to the knee joint, including fractures of the pelvic ring and acetabulum [11]. In particular, in one of the studies, the mental health status of a group of 32 adult patients with pelvic trauma was assessed 3, 6, and 12 months after the injury, showing a higher incidence of depression and PTSD in this group in the studied periods. The frequency of the described MHDs was higher than in the general population [12]. Also, one study of 148 men showed that higher VAS scores in patients with limb fractures were associated with the occurrence of PTSD [13].

A fracture in the pelvic area seems to be a disease that could potentially contribute to the development of depressive disorders. As a rule, it is a severe injury with accompanying injuries that drastically changes the current life situation [14,15,16,17]. This is associated with serious surgery, blood loss, and a long period of rehabilitation [12,18]. Cessation of professional activity is usually required for at least 2–3 months, but, in some cases, this period is much longer [19]. Several studies have already presented higher rates of depressive disorders in people with pelvic injuries. McMinn et al. and Martin et al. presented in their studies that almost 30% of the patients who suffered from pelvic injury are diagnosed with depression [12,20]. Those results indicate that the risk of developing depressive disorders due to pelvic fracture could be potentially almost ten times higher compared to the global population.

Due to the significant impact and relatively frequent occurrence of MHDs in patients with pelvic fractures, the aim the study was to assess the prevalence of depressive disorders in patients with pelvic fractures treated surgically at one trauma center in the period 2017–2022.

## 2. Materials and Methods

### 2.1. General Characteristics

**Scheme 1:**
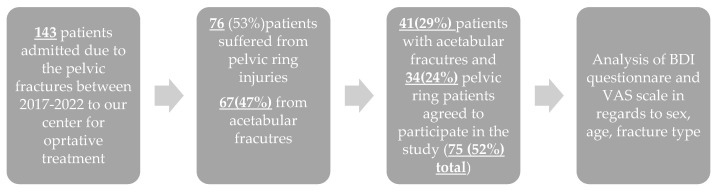
Presentation of enrollment process.

The study included patients operated on in our center in the years 2017–2022 due to a fracture of the acetabulum of the hip joint or a fracture of the pelvic ring. The inclusion criteria were as follows: pelvic ring or acetabular fracture treated operatively; admission and treatment in our institution between 2017 and 2022. Exclusion criteria: conservative treatment of pelvic fracture. Patients completed the surveys via the telephone. Interviews were conducted by two medical doctors of our trauma center in August 2024. The questionnaire included the Beck Depression Inventory (BDI). Additionally, patients were asked about chronic pain symptoms measured on the VAS scale, and subsequent analysis with the obtained BDI score was performed. Moreover, this scale was used as a retrospective assessment of the one-week period of time after the injury. The method of use results from the lack of other generally available and recognized tools for the retrospective assessment of depressive disorders in physical trauma patients. Scheme 1 presents the enrollment process.

#### Beck Depression Inventory, VAS Scale

Following questionnaire and interpretation are based on “An inventory for measuring depression, Archives of General Psychiatry, (4: 53–63).” Beck et al. Prior to the study, it had been translated to Polish. Patients provided only one answer to the given question. Each answer is associated with its score ranging from 0 to 3. Final BDI score is obtained by summing points from 21 questions. Following the BDI questionnaire, patients were asked to state the level of their pelvic pain using the VAS (Visual Analog Scale). Additionally, patients were asked whether they routinely use any analgesics. Questionnaires are provided in the Supplementary Materials. Definition of the post-traumatic chronic pain used in our study is according to the International Association of The Study of Pain: *“occurs 3 months after an injury or surgery, without taking into account infectious conditions and the severity of tissue damage; also, the person’s past pain is not important in this classification”.*

### 2.2. Ethics

Prior to the study, permission was obtained from the Nicolaus Copernicus Bioethics Committee in Torun (approval number KB 645/2022). The study was conducted in accordance with the Declaration of Helsinki guidelines for human experiments. Informed consent was obtained from all patients or their legal guardians to include them in scientific studies.

### 2.3. Statistical Analysis

All group comparisons and statistical analyses were conducted using Prism software version 10 (GraphPad Software, 225 Franklin Street, Fl. 26, Boston, MA, USA). Relationships between the studied parameters were evaluated using the Spearman’s rank correlation coefficient. Nominal variables were characterized with number of observations and their structure. Their comparison between the two groups was run with Pearson’s chi-squared test with or without Yates continuity correction depending on expected values for adequate contingency tables.

Numerical variables were described using basic statistics, i.e., mean and standard deviation in the case of normal distribution and median plus range for an abnormal distribution. Comparisons of the variables between groups were executed with Welch *t*-test for independent groups and Mann–Whitney U test depending on normality of their distribution. A *p*-value of less than 0.05 was considered statistically significant.

## 3. Results

**Figure 1:**
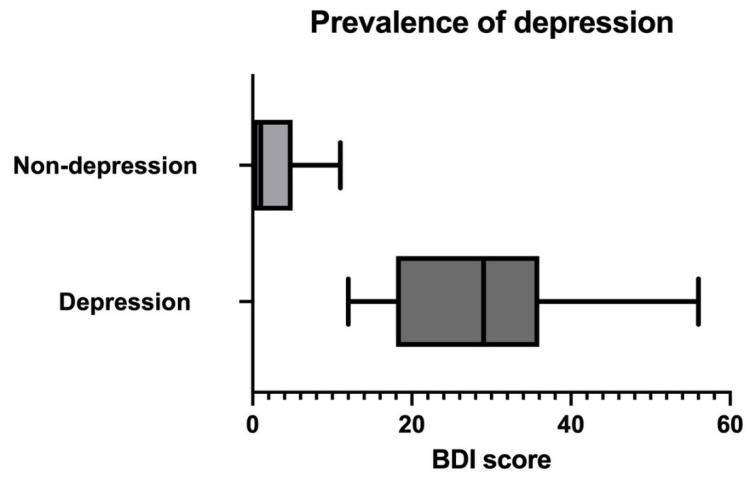
Prevalence of depression in studied population measured in BDI score. *p* < 0.0001.

**Figure 2:**
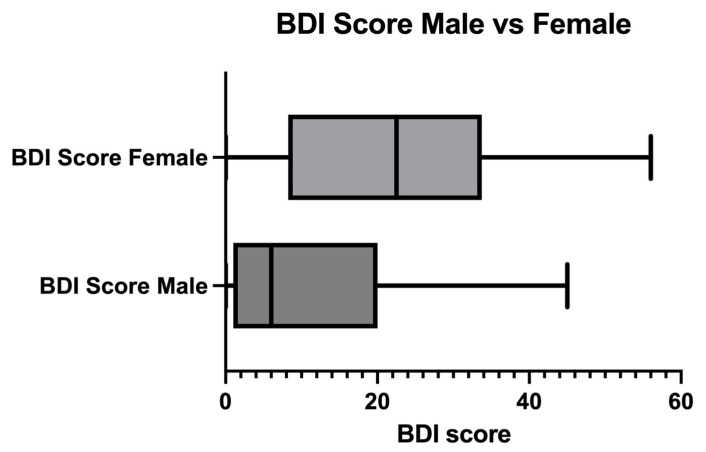
Comparison between BDI scores in male and female studied populations. *p* < 0.008.

The studied group consisted of 75 patients, 18 females (mean age 54 years old) and 57 males (mean age 53 years old). The mean duration between surgery and interview was 42 months. Moreover, 35 out of the 75 patients showed symptoms of depression (BDI score > 11), which is 47%, with an average score of 29 (*p* < 0.0001) (Figure 1; Table 1). Comparing the groups of women and men studied, on average, 72% of the women showed symptoms of depression (Figure 2). Women obtained an average BDI score of 23, which corresponds to moderate depression on Beck’s scale (*p* < 0.008) (Figure 2; Table 2). The average score in the group of men was 6, which does not suggest the presence of depressive disorders (Figure 2; Table 3). Furthermore, five out of the thirty-five patients (14%) with symptoms of depression admitted to undergoing psychiatric treatment following their injuries (Table 1).

**Figure 3:**
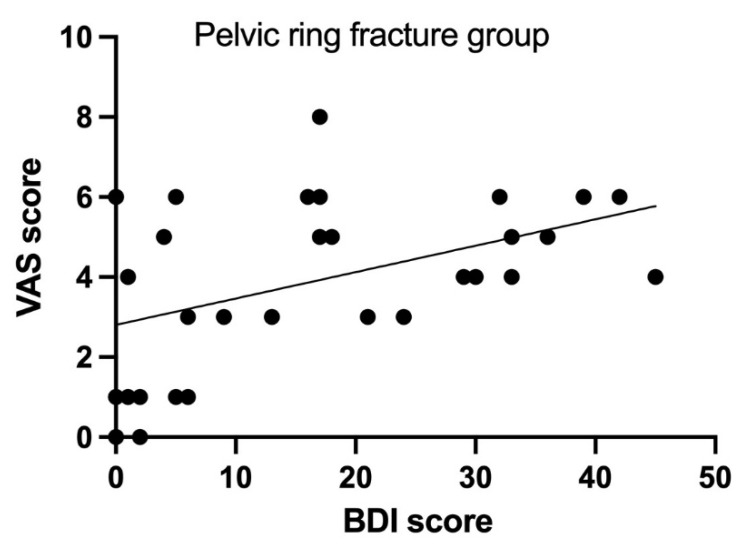
Correlation between VAS and BDI scores in pelvic ring fracture group. *p* < 0.005; r = 0.4013.

**Figure 4:**
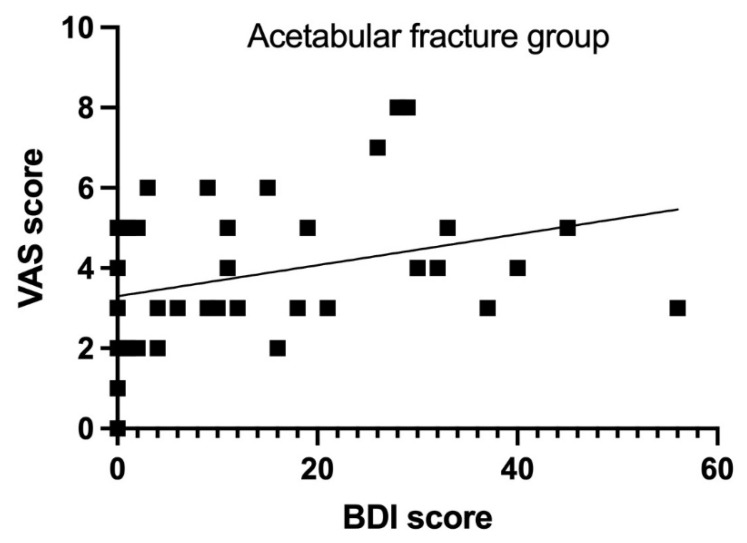
Correlation between VAS and BDI scores in acetabular fracture group. *p* < 0.01; r = 0.3813.

**Figure 5:**
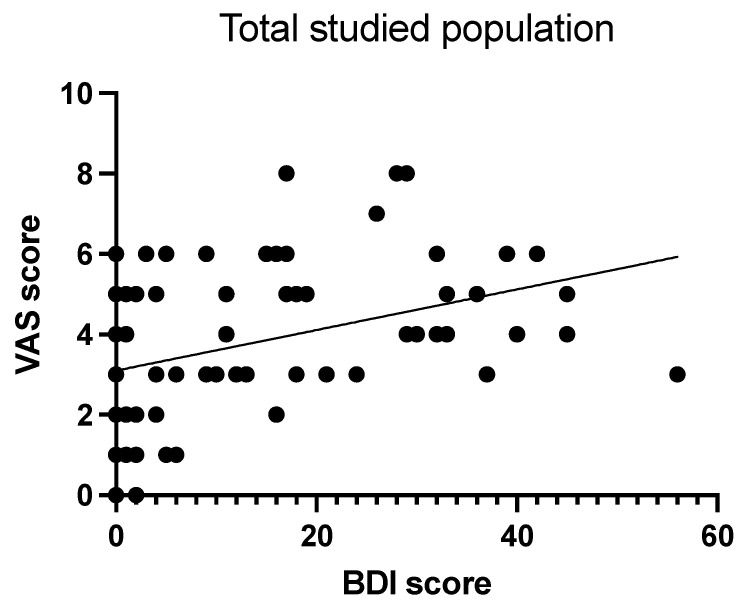
Correlation between VAS and BDI scores in total studied population. *p* < 0.0003; r = 0.4094.

A statistically significant correlation was also observed between chronic pain complaints and BDI score in the group of both pelvic ring (Figure 3) and acetabulum fractures (Figure 4). Seventy-two patients (96%) reported chronic pelvic pain following surgery that met the given criteria. An increase in the BDI score correlates positively with chronic pain complaints on the VAS scale (*p* < 0.0003; r = 0.4094.) (Figure 3, Figure 4 and Figure 5). Additionally, 71 of the 75 patients (94%) reported regular analgesics use due to pelvic pain following surgery (Table 2).

**Figure 6:**
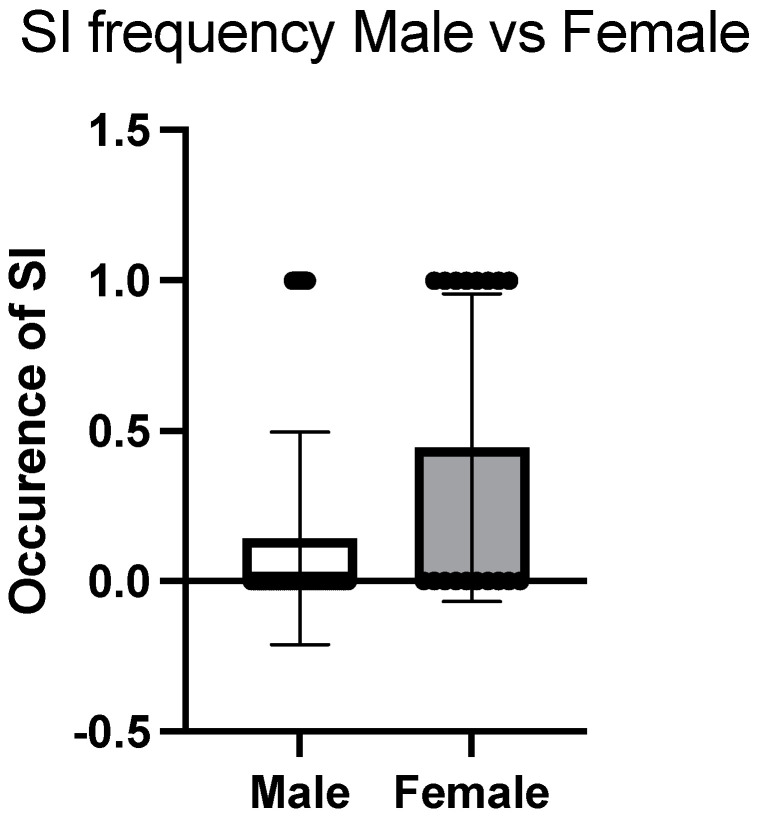
Frequency of suicidal ideation (SI) in studied population. *p* < 0.01.

What also has been proven is that women in our studied population reported suicidal thoughts more often than men, 44% vs. 14% (*p* < 0.01). It has been found to be statistically significant regardless of age and type of fracture (Figure 6).

**Figure 7:**
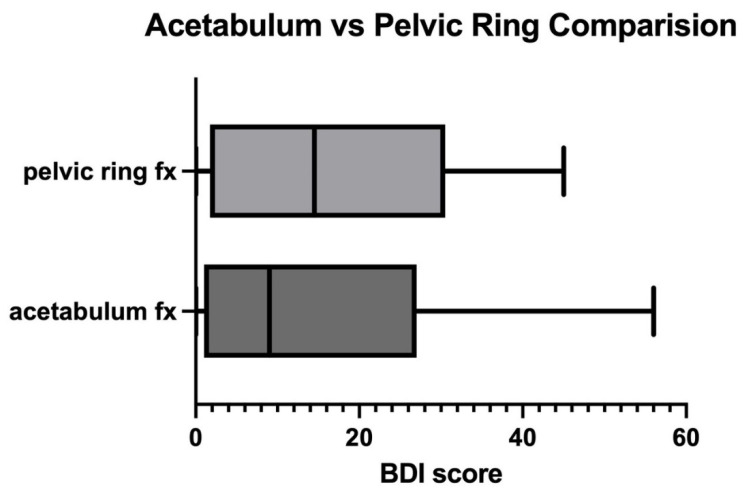
Comparison between mean BDI scores in acetabular and pelvic fractures. *p* < 0.3.

**Figure 8:**
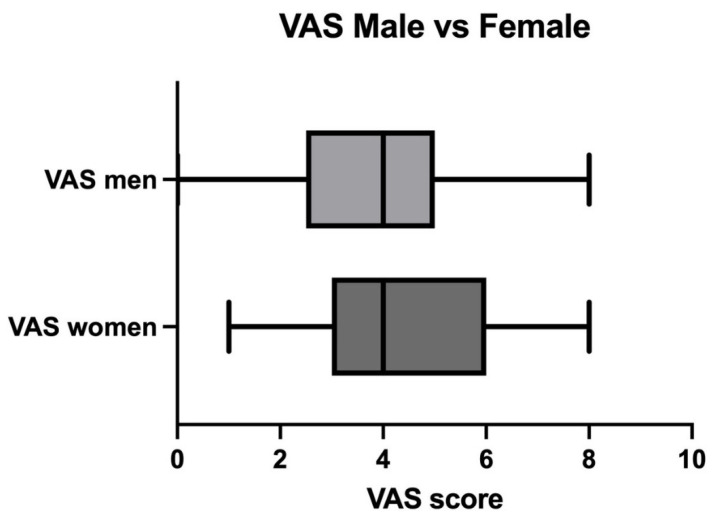
Comparison between VAS scores in male and female studied populations. *p* < 0.5.

No statistically significant differences were observed regarding BDI score when comparing the groups of acetabular fracture and pelvic ring fracture, 14 and 16, respectively (*p* < 0.3; Figure 7; Table 3). The differences in VAS mean scores between the male and female studied groups were found to be not statistically significant (mean score of 4 in both groups; *p* < 0.5; Figure 8). No statistically significant correlation was found between the occurrence of depression and the length of hospital stay. No statistically significant correlation was found between the occurrence of depression and the patient’s age (*p* < 0.2).

## 4. Discussion

### 4.1. Rate of Depressive Disorders

The results of our study indicate that patients who have sustained pelvic injuries, involving both acetabulum and pelvic ring fractures, are statistically significantly more likely to exhibit symptoms of depression compared to the general population (*p* < 0.0001) (Figure 1) [1]. The data presented here are derived from an analysis of patients treated at our center over the past 6 years, which serves as a reference facility in the region for managing patients with pelvic injuries. Among the 75 patients included in this study, 35 scored 12 or more points on Beck’s scale, suggesting the presence of depressive disorders in those individuals. This number accounted for 47% of the patients participating in the study (Table 1). Only five of the thirty-five patients (14%) with depressive symptoms received further psychiatric treatment following their injuries (Table 1). We suspect that a lack of a routine mental status evaluation in trauma patients could have an influence on such infrequent further psychiatric or psychological treatment. Our results confirm the findings reported by McMinn et al., where the authors documented the occurrence of depression in 31% of patients experiencing traumatic pelvic injuries [12]. Martin III et al. and Crichlow et al. also reported that symptoms of depression are more common among patients with severe pelvic ring fractures and orthopedic trauma [20,21]. The results of our study, as well as those previously mentioned, are consistent with the meta-analysis conducted by Muscatelli et al., which included data from 27 articles with a total of 7109 participants. This analysis indicates that the prevalence of depression among patients experiencing acute orthopedic trauma was 32.6% [22]. The authors of this study also noted that post-traumatic stress disorder (PTSD) was more frequently observed in the cohort of included patients [22].

### 4.2. Sex, Age, and Depression Rate

Although the proportion of depressive disorders following pelvic injuries was higher among women, it is important to note that such injuries occur significantly more often in men [23,24,25,26]. In our study, the prevalence of depression, as assessed by Beck’s scale, was observed to be 72% among women and 19.3% among men (Table 2, Figure 2). It is noteworthy that the average score for women was 23, which indicates a moderate level of depression (Table 2). In contrast, the average score for men was 6, suggesting the absence of depressive disorders (*p* < 0.008) (Table 2). It is not entirely clear whether the occurrence of depression in men with pelvic fractures is a more significant health problem than in women, but a substantial number of men may also require specialized psychological and psychiatric care.

In our study, the prevalence of depression, as assessed by Beck’s scale, was observed to be 72% among women and 19% among men (Table 3; Figure 2). It is noteworthy that the average score for women was 23, which indicates a moderate level of depression (Table 3). In contrast, the average score for men was 6, suggesting the absence of depressive disorders (*p* < 0.008) (Table 2). Our findings regarding the increased risk of depressive disorders in women are confirmed by the study of Schwartz et al., who analyzed data from 507 hospitals concerning mental disorders in patients with orthopedic injuries. Among these, the most frequently reported conditions were dementia (14.3%) and depression (6.9%). The group of patients most vulnerable to these mental disorders comprised white women over the age of 64 [27]. The lower incidence of depression reported in this study compared to ours may be related to the fact that the authors analyzed patients not only with pelvic injuries but also with lower-limb injuries. Winter et al., in a study involving 263,988 participants, demonstrated that the prevalence of mental disorders, including depression, is higher among individuals with pelvic and proximal femur fractures compared to those with fractures below the knee [11]. Williams et al., in a study involving 886 female participants, 296 of whom had sustained a fracture, conducted a 6-year follow-up to assess the occurrence of depression among the participants. Any kind of orthopedic fracture was associated with a threefold increase in the risk of developing depression during the study period, but only in the group of women over the age of 65. No association between fractures and the onset of depression was observed in women under 65 years of age [28]. Our findings, however, suggest that younger women also exhibit a higher incidence of depression associated with pelvic fractures. Nonetheless, we found no statistically significant correlation between patients’ age and their scores on Beck’s scale (*p* < 0.2). It should be emphasized that the conducted study confirms the more frequent occurrence of MHDs in women with pelvic fractures, which confirms the available literature data in this regard.

Fractures, especially those involving the femur, are associated with a higher prevalence of suicidal thoughts [29,30]. According to the data collected during this study, women were statistically more likely to experience suicidal ideation following a pelvic injury, with a prevalence of 44% compared to just 14% among men (*p* < 0.01).

### 4.3. Depression and Chronic Pain

We also observed that chronic pain is associated with a higher risk of depression. An increase in the Beck Depression Inventory score is positively correlated with chronic pain, as measured by the VAS scale (mean value in both groups 4; *p* < 0.01), although it is important to point out that the correlation coefficient is weak (r = 0.4094). This correlation was observed in both the group of patients with acetabular fractures and those with pelvic ring fractures. However, it is mandatory to highlight the routine use of analgesics by almost 94% of the studied population and the probable impact on the VAS score results (Table 2). The increased occurrence of depression in patients with chronic pain is likely influenced by changes in the nervous system at the molecular level [31,32]. Gerbershagen et al. conducted an analysis of 69 patients who sustained acetabular fractures, pelvic ring fractures, or a combination of both, focusing on the prevalence of chronic pain (38–90% depending on the fracture type) and its subsequent impact on quality of life. In those patients experiencing chronic pain, they reported, in line with our findings, an increased incidence of depression [33]. Additionally, these patients exhibited reduced physical and mental functioning and a higher frequency of comorbid injuries [17]. Other authors have also reported correlations between chronic pain and depression, noting more frequent occurrences of anxiety and poor sleep quality among patients suffering from chronic pain [34,35,36]. Based on these data, it appears that chronic pain may be one of the causes of MHDs in patients with pelvic fractures; therefore, effective treatment of patients with chronic pain is essential to prevent the occurrence of depressive symptoms in this population.

### 4.4. Impact of Length of Hospital Stay on Depression

Several studies have examined the impact of hospital length of stay on the prevalence of depression among various patient populations [37,38,39]. The results regarding the correlation between these variables have varied across different publications. In our study, we did not find any significant associations between hospital length of stay and the prevalence of depression (*p* < 0.2). One of the key findings of this study is also that the prevalence of depression during the follow-up period after injury does not differ significantly between the groups of patients with acetabular and pelvic ring fractures (*p* < 0.6).

### 4.5. Sexual Dysfunction and Chronic Pain

A very important issue that also needs to be addressed is sexual dysfunction (SD) after pelvic injury. In our studied population, 29 out of 75 (39%) reported reduced sexual activity via the BDI questionnaire. Those findings are in line with Rovere et al.’s systematic review concerning pelvic injuries and sexual activity. They presented that almost 40% of those patients who suffered from pelvic fracture reported SD, such as erectile dysfunction, orgasmic dysfunction, or dyspareunia [40].

## 5. Limitations

It should be noted that our study has several limitations. The analyzed group was relatively small, and all the participants came from our single trauma center. The study involves patients treated operatively due to pelvic fractures who agreed to participate in the study (51% of the patients, Scheme 1); therefore, there is no comparison to a group treated conservatively or any further information on those patients who did not consent to take part in this study. Information concerning their number, adherence rate, and clinical condition was not obtained. The lack of those data has a probable impact on the results. All the surgical procedures were performed by the same team, which may also influence the obtained results. Due to the lack of widely recognized tools for the retrospective assessment of depression occurrence in the post-injury period, we utilized the Beck Depression Inventory since it also provided essential information on sexual dysfunction and suicidal ideation. The time between trauma and the questionnaire was not analyzed in regard to its significance in depressive disorder development. It is worth mentioning that Birk et al. presented that people tend to report significantly higher levels of depression and suicidal thoughts for retrospectively reported depressive episodes compared to when they were not in depressive episodes [41]. Macchiarola et al. demonstrated that children with knee pathologies are prone to recall bias while performing the Pedi-IKDC in a retrospective manner [42]. Additionally, we supplemented it with the VAS scale to identify patients suffering from chronic pain, which, as we have shown, also affects the quality of mental health. Factors such as BMI, employment status, job changes, demotions, or dismissals post-fracture were not analyzed in this study. Those factors supposedly could have an impact on depressive disorder occurrence and suicidal ideation. The observation period took place during the COVID-19 pandemic, which, as indicated by other studies, may also be associated with a higher prevalence of depressive disorders [43,44,45,46]. According to some studies, the COVID-19 pandemic had an impact on the hospitalization process and length of hospital stay. Correia et al. presented that the lengths of postpartum hospital stays were observed to be shorter during the COVID-19 pandemic [47].

## 6. Conclusions

This study presents that pelvic trauma patients are at high risk of depressive disorders and suicidal ideation. In the studied population, women were more prone to suffering from depression and suicidal ideation compared to men. The overall prevalence of depressive disorders has been reported to be 47%, which is over ten times more compared to the global population [1]. Given our results, we believe that routine BDI questionnaire administration before discharge should be considered, with further follow-up in ambulatory care units. In the case of a result suggesting a depressive disorder, it would be highly advised to provide a referral to a psychiatrist. In the event of any evident metal status alteration during hospitalization, an immediate psychiatric consultation should take place. It is particularly important to provide psychiatric care for patients with a history of depressive disorders. Our results indicate that conducting further studies on the comorbidity of depression and pelvic fractures is warranted.

## Figures and Tables

**Table 1:** BDI scores of studied population with categorization in regard to the severity of depressive disorders and further psychiatric treatment of depression.

	No Depression	Mild Depression	Moderate Depression	Severe Depression
BDI Score	0–11	12–26	27–49	50–65
No. of patients	40 (53%)	15 (20%)	14 (19%)	6 (8%)
Psychiatric treatment	0 (0%)	0 (0%)	1 (7%)	4 (67%)

**Table 2:** Analgesics used by the patients with chronic pelvic pain.

Analgesic	Celecoxib	Diclofenac	Naproxen	Paracetamol + Tramadol	Ibuprofen
No. of patients	8 (5%)	19 (25%)	8 (5%)	16 (21%)	20 (27%)

**Table 3:** Comparison between mean BDI scores according to sex and type of fracture. *fx—fracture*; *M&F—male and female*.

	Male	Female	Acetabular Fracture	Pelvic Ring Fracture	M&F Overall
Mean BDI Score	6	23	14	16	23

## Data Availability

The data that support the findings of this study are not openly available due to privacy restrictions but are available from the corresponding author upon reasonable request.

## Supplementary Materials

The following supporting information can be downloaded at https://www.mdpi.com/article/10.3390/diseases13040105/s1, File S1: BDI questionnaire.
