# Supplementary material for: Prevalence of Depressive Disorders in Operatively Treated Pelvic Trauma Patients
Source: Diseases. 2025 Mar 31;13(4):105. doi: 10.3390/diseases13040105 (PMC12026098; doi:10.3390/diseases13040105)
Supplement: Supplementary file 1 [file diseases-13-00105-s001.zip › diseases-3513293-supplementary.pdf]

BDI questionnaire

1.

0 I do not feel sad.

1 I feel sad

2 I am sad all the time and I can't snap out of it.

3 I am so sad and unhappy that I can't stand it.

2.

0 I am not particularly discouraged about the future.

1 I feel discouraged about the future.

2 I feel I have nothing to look forward to.

3 I feel the future is hopeless and that things cannot improve

3.

0 I do not feel like a failure.

1 I feel I have failed more than the average person.

2 As I look back on my life, all I can see is a lot of failures.

3 I feel I am a complete failure as a person.

4.

0 I get as much satisfaction out of things as I used to.

1 I don't enjoy things the way I used to.

2 I don't get real satisfaction out of anything anymore.

3 I am dissatisfied or bored with everything.

5.

0 I don't feel particularly guilty

1 I feel guilty a good part of the time.

2 I feel quite guilty most of the time.

3 I feel guilty all of the time.

6.

0 I don't feel I am being punished.

1 I feel I may be punished.

2 I expect to be punished.

3 I feel I am being punished.

7.

0 I don't feel disappointed in myself.

1 I am disappointed in myself.

2 I am disgusted with myself.

3 I hate myself

8.

0 I don't feel I am any worse than anybody else.

1 I am critical of myself for my weaknesses or mistakes.

2 I blame myself all the time for my faults.

3 I blame myself for everything bad that happens.

9.

0 I don't have any thoughts of killing myself.

1 I have thoughts of killing myself, but I would not carry them out.

2 I would like to kill myself.

3 I would kill myself if I had the chance.

10.

0 I don't cry any more than usual.

1 I cry more now than I used to.

2 I cry all the time now.

3 I used to be able to cry, but now I can't cry even though I want to.

11.

0 I am no more irritated by things than I ever was.

1 I am slightly more irritated now than usual.

2 I am quite annoyed or irritated a good deal of the time.

3 I feel irritated all the time.

12.

0 I have not lost interest in other people.

1 I am less interested in other people than I used to be.

2 I have lost most of my interest in other people.

3 I have lost all of my interest in other people.

13.

0 I make decisions about as well as I ever could.

1 I put off making decisions more than I used to.

2 I have greater difficulty in making decisions more than I used to.

3 I can't make decisions at all anymore.

14.

0 I don't feel that I look any worse than I used to.

1 I am worried that I am looking old or unattractive.

2 I feel there are permanent changes in my appearance that make me look unattractive

3 I believe that I look ugly.

15.

0 I can work about as well as before.

1 It takes an extra effort to get started at doing something.

2 I have to push myself very hard to do anything.

3 I can't do any work at all.

16.

0 I can sleep as well as usual.

1 I don't sleep as well as I used to.

2 I wake up 1-2 hours earlier than usual and find it hard to get back to sleep.

3 I wake up several hours earlier than I used to and cannot get back to sleep.

17.

0 I don't get more tired than usual.

1 I get tired more easily than I used to.

2 I get tired from doing almost anything.

3 I am too tired to do anything.

18.

0 My appetite is no worse than usual.

1 My appetite is not as good as it used to be.

2 My appetite is much worse now.

3 I have no appetite at all anymore.

19.

0 I haven't lost much weight, if any, lately.

1 I have lost more than five pounds.

2 I have lost more than ten pounds.

3 I have lost more than fifteen pounds.

20.

0 I am no more worried about my health than usual.

1 I am worried about physical problems like aches, pains, upset stomach, or constipation.

2 I am very worried about physical problems and it's hard to think of much else.

3 I am so worried about my physical problems that I cannot think of anything else.

21.

0 I have not noticed any recent change in my interest in sex.

1 I am less interested in sex than I used to be.

2 I have almost no interest in sex.

3 I have lost interest in sex completely.

**BDI Score**	**Interpretation**
**0-11**	No depression
**12-26**	Mild depression
**27-49**	Moderate depression
**50-65**	Severe depression

VAS scale

How would you describe the level of your pelvic pain?

No Pain ***1 2 3 4 5 6 7 8 9 10*** ***Severe Pain***
